# The Hospital. Nursing Section

**Published:** 1904-08-20

**Authors:** 


// The Hospital.

**Nursing Section.**

Contributions for this Section of "The Hospital" should be addressed to the Editor, "The Hospital" Nursing Section, 28 & 29 Southampton Street, Strand, London, W.C.

No. 934.—Vol. XXXVI. *SATURDAY, AUGUST* 20, 1904.

### Notes on News from the Nursing World.

### QUEEN ALEXANDRA'S MILITARY NURSING SERVICE.

We are officially informed that the following appointments have been made to Queen Alexandra's Imperial Military Nursing Service. To be Sister :—Miss M. Smith, posted to Netley. To be Staff Nurses :—Miss F. A. Dawson, posted to Caterham ; Miss F. G. P. de Stourdza-Zrinyi and Miss G. M. Smith, posted to Woolwich ; Miss H. M. Drage, Miss E. M. Keays, Miss E. L. McAllister, Miss E. M. Perkins, and Miss M. E. Wilkin, posted to Netley. Miss E. L. C. Crowe, staff nurse, has resigned her appointment. The following changes of station have been made. Matrons :—Miss C. M. Chadwick, R.R.C., to Harrismith, Orange River Colony ; Miss E. Ferguson, to Wynberg, Cape Colony ; Miss M. Russell, R.R.C., to Standerton, Transvaal ; Miss M. Thomas, to Pretoria. Sisters :—Miss C. Anderson, to Barberton ; Miss E. A. Cox and Miss D. F. Palmer, to Potchefstroom ; Miss M. E. Harding, Miss A. C. Jacob, and Miss M. G. A. Warner, to Wynberg ; Miss M. G. Hill, R.R.C., and Miss F. M. Hodgins, to Pretoria ; Miss J. Hoadley, R.R.C, and Miss E. M. E. Todd, to Maritzburg ; Miss M. R. Makepeace, to Kroonstad, Orange River Colony ; Miss E. J. Martin and Miss M. Steenson, to Standerton ; Miss A. Nixon and Miss A. R. Rose-Innes, to Middelburg ; Miss D. I. Rickards, to Bloemfontein ; Miss S. I. Snowdon, to Krugersdorp ; and Miss L. M. Todd, to Mooi River. Miss E. Barber and Miss G. Knowles have been confirmed in their appointments as staff nurses. The following staff nurses have been promoted to be Sisters :—Miss M. M. Bond, Miss A. F. Byers, Miss E. M. Denne, Miss M. Kendall, Miss S. B. Lanyon, Miss E. M. Pettle, Miss C. G. Stronach, and Miss A. A. Wilson.

### THE NEW NURSES' HOME AT NETLEY HOSPITAL.

Practically, the new quarters of the nursing staff at the Royal Victoria Hospital, Netley, constitute a handsome nurses' home. They are in the centre of the magnificent block of buildings stretching over the ground facing the sea. Part of the quarters is taken from the portion of the hospital which at one time was set aside for the surgeons on probation, who now study in London and therefore do not require the rooms ; and the other part consists of the former sick officers' quarters, and various offices adjoining. The matron has her own rooms adjacent to those of the sisters, and each sister and staff nurse has a comfortable bedroom, as well as the use of a general sitting-room and dining-room. More bath-rooms have been added, quiet rooms for the night staff, a convenient kitchen, and servants' hall. The new quarters were badly needed, the old ones being cramped and uncomfortable in every way. There was only a single bath-room for the whole household, and the sisters had only cubicles for half the staff. These were very small, and there was no dining-room. The staff at Netley, as reorganised, is nearly completed, and will comprise the matron, the home sister, 10 sisters, and 16 staff nurses. Now that the conditions have been so greatly improved, there is no lack of candidates for appointments to Queen Alexandra's Imperial Military Nursing Service.

### RYAN v. THE CROWN.

Miss Ryan, who failed in her action against the Crown the other day, informs us that she does not regret the course she pursued, although it has involved her in very heavy expenses. She further urges that her case failed on the technical point of law, and that it "was not the pleasure of the Crown that she was compulsorily retired without any reason." In addition, Miss Ryan states that her solicitor informs her that "if the action had been an ordinary civil one, it must have been decided in her favour." Possibly, but it was not an ordinary civil case. Miss Ryan's pension is, it appears, £32 4s., and not £34 per annum as stated.

### THE SUPPLY OF JAPANESE NURSES.

A striking proof of the importance which the Japanese attach to the services of female nurses is given by the arrangements made for tending the sick and wounded in a passenger vessel which has lately been converted into a hospital ship, with its saloons fitted with swinging cots and its cabins used as operating and dressing rooms. The vessel accommodates only 180 patients, and no fewer than 50 nurses, or one to every three or four patients, have been put on duty. This also confirms the statement that the supply of Japanese nurses is adequate without recourse to volunteers belonging to other countries. All sorts of places appear to be utilised by the Japanese for the wounded, and a number of Russian prisoners—officers and men—are quartered in a palatial old castle. They are placed in rows on the floor with the exception of the small number seriously hurt, who are in ordinary beds. The wounded prisoners and their nurses, who are clad in white and wear sandals, get on exceedingly well together.

### MARRIAGE OF AN ARMY RESERVE SISTER.

On Tuesday last week Miss Lotta Schröder, lately a member of the Army Nursing Service Reserve, was married to Captain R. H. Kendle, late Wilts Regiment, at St. Martin's Church, Brighton. Miss

Schröder was trained at the Royal Infirmary, Liverpool, where she held the post of sister for four and a half years, resigning the post in order to go to South Africa. During the year she served in the war she was attached to No. 12 General Hospital at Springfontein, Orange River Colony.

### A GUY'S NURSE DROWNED NEAR MAIDSTONE.

On Tuesday morning a very painful sensation was caused at Guy's Hospital by the announcement that Miss Elise Riley, a member of the nursing staff, had been drowned at Allington, a mile from Maidstone, on Monday afternoon. Miss Riley, whose engagement to be married to Mr. C. Pye-Smith, of Guy's, was only announced a fortnight ago, was making the trip to Maidstone in one of the Government launches, accompanied by Mr. Pye-Smith, her mother, and other relatives. On reaching Allington Castle, on her return journey, Miss Riley was walking the narrow deck of the boat when she was suddenly thrown into the water. The launch was going about seven miles an hour, and before it could be stopped Mr. Pye-Smith and two stokers dived overboard. Miss Riley was caught by one of the stokers, but the man being hampered by his clothes was obliged to release his hold, and she sank. Her body was subsequently recovered and artificial respiration was attempted without success. Miss Riley was a daughter of an officer in the Royal Naval Reserve, at Chatham, and was only 25 years of age. Her sad death, just when life seemed to be fairest for her, is keenly regretted by everyone at Guy's Hospital and the utmost commiseration is expressed for the bereavement sustained by her relatives and by Mr. Pye-Smith.

### NORTH STAFFORDSHIRE NURSES AND THEIR HOME.

The appreciation by the nurses of the North Staffordshire Infirmary of their handsome and well-equipped new Home, which is fully described in another column by our commissioner, was attested in a striking manner by an event that lately took place. Presentations to nurses are often recorded, but this was a presentation to a friend of nurses, who has shown his interest in their welfare in the most practical manner. The presentation took place in the Nurses' Home at Hartshill, and was made by the nursing staff to Mr. A. F. Coghill, with whose help during his term of office as president the Home was erected, and by whose munificence it has been considerably endowed. The present consisted of a memorial scroll, signed by all the nurses who first entered the Home, enclosed in a silver casket. The function itself was short but interesting. Tea having been partaken of in the hall, which was decorated with flowers and ferns for the occasion, the presentation followed. It was made to Mr. Coghill in the presence of Mrs. Coghill and her daughter, and Miss Creagh. Mr. J. W. Phillips, a much valued member of the committee, to whom the addition of open-air wards is due, was also present, and considerable enthusiasm prevailed.

### NURSES AS DISPENSERS.

It has been incorrectly stated that the Local Government Board were asked to sanction the appointment of a nurse to act as dispenser at the Edmonton Workhouse. The facts of the case are that a nurse was assisting the medical officer of the workhouse in dispensing, but she was not appointed by the guardians to do the work, was not qualified, and the Local Government Board objected. The objection is, of course, a very proper one. A nurse who does not possess a qualifying certificate ought not, under any circumstances, to be permitted to dispense, or to assist in dispensing, and we hope that the Edmonton Guardians have peremptorily put a stop to an arrangement which should not have been made.

### UNION HOSPITAL, NEWCASTLE-ON-TYNE.

A very interesting function recently took place in the Union Hospital, Newcastle-on-Tyne, the occasion being the presentation of certificates to four probationer nurses who had completed their courses and successfully passed the examination. The hospital committee, to mark their appreciation of the event, invited a number of citizens interested in the institution to be present. Mr. Joseph Curry, chairman of the committee, presided, and gave a prize to Nurse Neylon, who obtained the highest number of marks at the final examination. The other three successful candidates were Nurses Dunn, Hugill, and Ormiston, the latter receiving a prize from Miss Clark, superintendent of the nursing staff, for having headed the terminal examinations most frequently. Sir George Hare Philipson, M.D., in presenting the certificates to the nurses, said an encouraging word to each recipient, and afterwards addressed the company present, expressing his great satisfaction with the hospital and its surroundings. Subsequently the guests were entertained to tea by Dr. Whillis, the principal medical officer.

### HONG KONG NURSING INSTITUTE.

Some weeks ago we mentioned that Sir Paul Chater had promised a thousand dollars a year to the Hong Kong Nursing Institute, and that in consequence of his generosity, and of other support promised, it was proposed to build a home for the Nursing Institute. At a general meeting of the supporters of the Institute, held last month, there was, however, a strong manifestation of hostility to the scheme; and ultimately, instead of passing a resolution authorising the committee to commence the building being passed, an amendment to the effect that the Hong Kong Government be approached with a view to set apart a sufficient portion of the Victoria Hospital to house the nurses and matron was adopted by a large majority. Whether the Hong Kong Government, in whose hands the matter rests, will consent to permit the Victoria Hospital to be used for the purpose of accommodating the nursing staff of the Institute, remains to be seen. As the latter is carried on for the benefit of well-to-do people, who can afford to pay for the services of a nurse, we agree with one of the speakers at the meeting who urged that it should be run on sound financial lines.

### NURSES FOR THE HOP-PICKERS.

The hopping season is close at hand, and by the end of next week there will be some 40,000 people at work in the hop gardens of Mid and West Kent. As on former occasions, the presence of trained nurses on the spot in the season is greatly desired, and the Hop-Picking Mission Committee, who carry on a very useful work during the period, are appealing for help of this kind. Last year the nurses at Farleigh West, Chilham, Malling East, Mereworth, Nettleshead, Paddock Wood, Peckham East, Teston, Tudeley, Five Oak Green, and Wateringbury, rendered most valuable service, while the Little Hoppers' Hospital, at the last-named place, with a sister in charge, was a great boon. Its necessity may be judged by the fact that there were 10 in-patients, and that 288 attendances of out-patients were recorded. Nurses who feel that they would like to render assistance should write to the hon. secretary of the committee, Rev. Francis G. Oliphant, Teston Rectory, Maidstone.

### NORFOLK AND NORWICH STAFF OF NURSES.

The thirty-seventh annual report of the Norfolk and Norwich staff of hospital-trained nurses, which has just been issued, contains some interesting particulars. The staff now consists of 48 surgical, medical, and monthly nurses, and one probationer in course of training. During the year nurses were supplied to 389 cases, representing 1,773 weeks of nursing. Most of these cases were nursed at considerably reduced fees, and 38 were without any fee. The branch home, formally opened in November, has been found a great convenience. Miss Blanche Sykes, who was appointed matron in April, in succession to Miss Creighton, is maintaining the excellent traditions of the organisation, and it is hoped that the example of Thorpe Hamlet, in subscribing a substantial sum for the services of one of the nurses from the home, will be followed by other parishes.

### LECTURES IN PRISON.

A short time since an interesting experiment was tried at Wormwood Scrubbs Prison of selecting a certain number of prisoners and allowing them to attend lectures. But the results were so disappointing that it is a matter for congratulation that someone was willing to make a second attempt, which has been successful. Miss C. Smith-Rossie, a lecturer under the Hampshire County Council, obtained permission from the authorities of Portsmouth prison to deliver lectures to the female prisoners on nursing, sanitation, and the care of children. She found that her remarks were listened to with "an almost disconcerting intensity of interest." Permission to attend the lecture is given only as a reward for good behaviour, which in itself tends towards improvement. Following this innovation, lecturers and nurses who have time to spare, and are not in need of remuneration, may care to offer their services for a course of lectures in any town where there is a large prison, and thus help to afford edification to, as well as to brighten the lives of, their sisters who have fallen by the way.

### THE "BARNSLEY HOME FOR NURSES."

There was opened last week, in connection with the Stockport Sick Poor and Private Nursing Association, the "Barnsley Home for Nurses," a modest addition to its public institutions of which the town may well be proud. The home, as the name denotes, is a memorial building, and was erected with money left for charitable objects by the late Miss Barnsley. Her trustees having decided to found it in commemoration of the Barnsley family, a suitable residence was purchased, adopted, and decorated at a cost of £1,000. The rooms are large and lofty, and the house is in every way suitable for the purpose to which it is devoted. It is near to the large mass of the population, and judging by the speeches delivered at the opening ceremony, the gift is likely to act as an incentive to the Stockport people to support the work of the Association.

### A WARNING TO CYCLISTS.

Like other people, nurses are sometimes foolhardy, and will not observe the most simple precautions for their protection against accidents. At the summit of the racecourse hill at Scarborough there is a danger signal, in spite of which cyclists persist in riding down and risking the sharp curve at Bell's Corner. The other day a nurse who is on a visit to the town rode down the hill, notwithstanding the caution, and, losing control of her machine, she was pitched off so heavily that her collar-bone was broken, and her hands were badly bruised. She was put in a passing cab and taken to the hospital, a sad ending to her holiday. Nurses who have to take care of other persons ought to be able to also take at least ordinary care of themselves.

### POOR LAW NURSES AND MIDWIFERY.

Last week we mentioned that four of the nurses at Ashton-under-Lyne Workhouse Infirmary had obtained the certificate of the London Obstetrical Society, and now we hear that Nurses Morrison, Sheppard, Greenhalgh, and Rogier, of the Grimsby Union Infirmary, have passed the examination of the society. These and other successes in different parts of the country justify the conviction that Poor Law nurses as a class intend to take full advantage of the opportunities offered under the Midwives Act of strengthening their own position by increasing their qualifications.

### A HOLIDAY QUESTION.

At the last meeting of the Dorchester Guardians a very important question was raised. The head nurse having been granted a fortnight's holiday, a proposal was made to engage a trained nurse during the period; this two members of the Board opposed on the ground of expense. Thereupon, the master of the workhouse stated that there were two serious cases in the infirmary calling for careful attention; and Canon Hankey declared that he would vote for the engagement of a trained nurse, because he was a guardian of the poor, and because if they engaged an untrained nurse, and anything happened, the Dorchester Guardians would be gibbeted in every newspaper in England. We are glad to say that the proposal was then agreed to by a large majority. It is, of course, the duty of Boards of Guardians to protect the pockets of the ratepayers, as well as to look after the interests of the poor. But the latter should always stand first, and the employment of an untrained nurse in the place of a trained one, while the latter was away for a holiday, would have been a perilous piece of economy which no immunity from evil results could possibly have justified.

### The Nursing Outlook.

"From magnanimity, all fear above;
From nobler recompense, above applause,
Which owes to man's short outlook all its charm."

### THE ROLL OF HONOUR.

It would be possible to draw up a roll of 365 heroines who have served well the sick and suffering, and to have before us a daily ensample of women's work as nurses. As a rule we are apt to dwell too much on the one or two names that are known to fame, and not to seek diligently for the stories of the humble and the meek. And yet what a glorious roll would be a calendar of the servitors of the sick! It would extend from Dorcas to Agnes Jones; from the daughter of Damophilus to Dorothea Lynda Dix. It would cover all countries, all castes, all creeds.

There is the story of Santa Chiara—"to whom Saint Francis sent many sick persons," says the 33rd chapter of the "Fioreth," a story in which few take any interest, in spite of the Fransciscan revival. Indeed, so careless are modern writers that Abbot Gasquet, in his book just published on "English Monastic Life," calls St. Clare the sister of St. Francis; and Miss Echenstein, in her "Women under Monasticism," speaks of the "narrow interests" to which St. Francis confined the nuns of St. Clare. Those who have visited the little convent just without Assisi and seen the relics there guarded, and heard and studied the stories of St. Clare, know how far from narrow her life was, and how her actions on two occasions saved the town.

Then there is the Princess Agnes of Bohemia, to whom St. Clare wrote and sent gifts. Princess Agnes refused marriage and accepted poverty; she founded a hospital at Prague which exists to this day, and, according to a contemporary account, "she spread soft beds for them, she carefully removed all that could distress eyes and nose, she prepared food with her own hands, that the sick might be freed from ill, pains diminish, illness yield, and health return." The story of another princess, St. Elizabeth of Hungary, is known to us all through Kingsley's splendid poem, "A Saint's Tragedy."

But to turn to lowlier walks in life. There is the story of the servant who, under the Abbé le Pailleur, in 1840 founded the Little Sisters of the Pon at St. Malo. Jeanne had nothing of her own, and she was surrounded by the aged sick, whose miseries tore her heart out. She was herself over 40 years of age. One day she took up her basket in one hand, and with the other clasping the cross she wore round her neck, she went out and begged from door to door for her poor folk. The story is told that once some man, wearied by her importunities, smacked her face. "Yes," said Jeanne, "that is all right, that is what I deserve; but now, what are you going to give me for my poor folk?" How many of us remember this story when we see the cart of the Little Sisters of the Poor driving about collecting broken food and alms.

Another story from France has the same beautiful simplicity: Sister Maria Theresa, superior of the Order of Sisters of Mercy at Tongking, was some 15 years ago decorated with the Legion of Honour in the presence of the assembled French troops. She had nursed for the army in Syria, China, and Mexico, and once during the Franco-German war she picked up a smoking shell which was thrown into the field-hospital and carried it out into a meadow; the shell burst just as she dropped it, and the sister was severely wounded. She was very nervous and depressed during the affixing of the Legion of Honour, and directly the Governor had pinned on the cross she asked, "And now may I return to my wounded soldiers?" More than 30 nurses have been made Chevaliers of the Legion of Honour in France, most of them nuns. The first was Mme. Biget, afterwards known as Sister Martha, who was decorated by Napoleon in 1815.

In our own country we might well remember the work done during the small-pox epidemic of 1871 by the Anglican sisters of East Grinstead, and the Sisterhood of All Saints, which for many years nursed two or three of the big London hospitals. It is interesting to note that Dr. Neale, who founded the East Grinstead sisterhood, considered three months sufficient training in nursing. If we turn to our own colonies, both Canada and India can supply us with stories of nursing heroism in the face of plague and famine, of sickness, hardship, and death. And there is the story of St. Rose of Lima, in Peru, and others to whom our civilisation and dependence on appliances and society were unknown.

Quite lately we have referred to Anna Stolberg and the deaconesses of Germany, but there are also the nursing deaconesses of Switzerland and elsewhere. And there are the nurses who went out to join Sir William MacCormac at Sedan; they ate dead horse and drank dirty water without grumbling, which is surely heroism enough to cause us to recall their names—Miss Pearson, Miss Macloughlin, Miss Barclay, Miss Neligan, and Mrs. Mason.

Our lines have fallen in pleasant places, and sometimes, merely to prove our womanhood and our devotion, we could wish that hardships were more common. The tradition of heroism must not be allowed to die out of the nursing world. There are few enough walks in life in which women have excelled, there are few enough rolls of honour on which their names can appear.

### Lectures upon the Nursing of Infectious Diseases.

By F. J. Woollacott, M.A., M.D., B.S.Oxon, D.P.H., Senior Assistant Medical Officer, Park Hospital, Metropolitan Asylums Board, Hither Green.

### LECTURE III.—INFECTIOUS NURSING IN PRIVATE.

The treatment of infectious diseases may be considered under two heads: First, the care of the sick, and, secondly, the prevention of infection. In practice the two are closely allied and every competent nurse should be familiar with both aspects. She should possess a knowledge of the main features of the disease, the common complications, and the signs of danger, and should, of course, be thoroughly skilled in all the methods of treatment usually left to be applied by her. In addition, she must be acquainted with the precautions necessary to guard against the spread of infection. General hospitals afford but few opportunities of gaining practical instruction in these matters, so that it is becoming the custom for nurses at the end of their training to join the staff of a fever hospital for a time.

In this lecture we shall consider the management of infectious diseases in general, with special reference to nursing in private houses. Sufferers from these diseases even more than others must be placed in thoroughly hygienic surroundings. Fresh air, warmth, and light are essential, and, if possible, a large, airy, bright, and well-ventilated room should be chosen. Stuffy rooms are an abomination, for in them the infection becomes concentrated, the vitality of the patient is depressed, and the risk to the nurse increased. Ventilation, to be efficient, must furnish a constant and ample supply of pure air, at the same time removing the air vitiated by respiration or otherwise. By so doing it carries off, or at all events largely dilutes, the poisons produced by the disease. In ordinary dwelling-rooms the chief means of ventilation are the windows and the fireplace. The former should be constantly open, and a fire kept burning in order to create an upward current of air in the chimney. To avoid the draught that may result from an open window the following device is useful: The lower sash is raised a few inches, and the gap closed with a piece of wood of suitable size. The external air then passes between the two sashes and enters the room in an upward direction, and does not strike directly upon the occupants. Sunshine may be admitted freely, and it must be remembered that a dull room means a dull patient. In most cases a temperature of about 62° F. is to be maintained.

As regards the general care of the patient no special rules are necessary. Cleanliness is of course of supreme importance, and all soiled articles should be removed as soon as possible. The food should be simple and easy of digestion, especially if there be any pyrexia.

In preparing a room for the reception of a case of infectious disease the first essential is to admit only articles of furniture that can be subsequently disinfected easily and without danger. A metal bedstead is the best, and such things as wardrobes and chests of drawers should be avoided as much as possible. Leather covered and elaborately padded chairs, pictures, and valuable ornaments are unsuitable, and should not be allowed. The next thing is to shut off communication as far as possible with the rest of the house, and with this object in view the room chosen is usually at the top or in an out-of-the-way part. An ideal arrangement would be a room entirely detached or connected with the house by means of a corridor with windows on each side, so that a cross-current of air could be maintained. This, unfortunately, is rarely available. The ordinary plan is to hang outside the door of the sick-chamber a large sheet in the form of a curtain reaching down to the floor. It must be kept constantly wet with some disinfectant solution, the object being that the infected dust from the room may adhere to it and not be blown into the house. From time to time it is advisable to purify it by boiling.

During the course of the case the following routine should be observed. Discharges from the nose and throat of the patient should be received on pieces of lint or rag, and these, with all dressings, should be immediately burnt. Remnants of food are best treated in the same way; they should never be taken from the room and consumed by other people. Dirty bed clothes, personal and table linen, should be put into a large vessel, such as a foot-bath, filled with a disinfectant solution, and afterwards boiled. They must never, under any consideration, be sent to a laundry until this has been done. Soiled articles are to be put at once into a covered pail and well soaked in a 5 per cent. carbolic solution. They may then be washed by the nurse and subsequently boiled. In enteric fever cases the stools and urine should be mixed with the same solution before they are allowed to enter the drains. The cleansing of crockery, etc., should be undertaken by the nurse herself. Letters written in the sick-room must not be sent through the post without efficient disinfection. Visits to the patient of the other inmates of the house should, as far as possible, be discouraged and prevented. Often, however, they will not submit entirely to such a restriction; so that in this particular, at least, strict isolation can rarely be enforced. Children, at all events, should never be allowed in the room.

At the conclusion of the case steps must be taken to effect a thorough disinfection of the patient, the sick-room, and its contents. The patient should take a hot bath and have his head shampooed. In hospitals a special set of rooms is used for this purpose. They are three in number, the middle one containing a bath. In the first the patient leaves his infected clothing, in the second he is washed, and in the third he puts on clean clothes. The contents of the sick-room are next dealt with. All useless and worn-out articles should be burned. Linen and cotton articles should be boiled, while the blankets, mattress, pillows, carpet, curtains, and books should be sent to the steam disinfector. The bedstead, tables, and chairs should be thoroughly scrubbed in the open air with soap and water, and then washed with a 5 per cent. carbolic solution. Such things as crockery and ornaments may be treated in the same way, or, better still, boiled. Finally, the room itself needs attention. This is the work of the sanitary officials. The wall-paper is stripped off and burnt, and the room fumigated. The usual plan is to make it as far as possible air-tight by pasting paper over the fireplace, ventilators, and windows. A quantity of sulphur is then ignited and left burning, and the door is shut and sealed up. After 24 hours the room is reopened and aired for a day or two. The walls and floor are then thoroughly cleansed with a disinfectant, the ceiling whitewashed, and the walls repapered. The whole process may seem somewhat complicated, but with the help of intelligent sanitary officials it is easily accomplished.

While attending upon a case of infectious disease a nurse always incurs a certain amount of risk, and she should endeavour, as far as possible, to safeguard herself. It is as much her duty to do this as it is the duty of the soldier to take cover in battle. Needless exposure to danger is always foolish. In the first place, she should take pains to keep in good general health; for if she becomes seedy or "run down," she will be more susceptible to the attacks of disease. A good appetite and daily open-air exercise are her best friends. In the second place she should be familiar with the mode in which infection leaves the patient, and so be able to take suitable precautions. This varies considerably in different diseases. In some, of course, the virus is given off more or less from all parts of the patient's body, and becomes diffused throughout the room; but even in these cases something may be done. In scarlet fever and measles, for instance, much of the infection originates in the throat and nose, and the discharges from these, with the sputum, are a special source of danger. In diphtheria, almost all the infection is spread in this way. It is a wise precaution, therefore, for the nurse not to bring her face too close to that of the patient, especially if he be coughing, and to remove and burn all discharges at once. If by accident any discharge should be coughed into her face, it should be washed off at once. The eye especially should be thoroughly bathed with warm water and boracic lotion. A sore throat should receive medical attention at once. The specific organisms of enteric fever are for the most part given off in the excreta; and in the same disease the pus from abscesses, especially if in connection with diseased bone, is often very virulent. The poison of erysipelas again often enters by means of scratches and small wounds. If the nurse have any such on her hands they must be carefully covered up. Plague may be acquired in much the same way, while in the pneumonic form of that disease the chief danger resides in the expectoration. Unfortunately, in spite of every precaution, the nurse is occasionally infected. Against only one disease can she be rendered invulnerable, and that is, curiously enough, the most infections of all, viz., small-pox. In this instance recent and efficient vaccination is a sure protection.

During her attendance on the case the nurse should be very careful not to carry about infection. Her infected clothing must be kept apart from her other things; and, of course, before going out she must change her clothes. If she is going to pay a visit she should first take a bath. At the end of the case all her infected clothing and property must be as thoroughly treated as are those of the patient. Neglect of these precautions may be disastrous to her next case; or to her own personal friends.

### The Arrival of No. 1.

BY A MATERNITY NURSE.

The hour is 2 A.M. when Mr. Younghusband becomes aware of being poked in his back by his wife Belinda, who is at the same time imploring him to get up, and when he does so and gropes round for the matches, tells him in a frightened voice "I am afraid dear you will have to go for the nurse!" Hastily he scrambles into his clothes, the night is cold, and that and the "unknowns" of the coming event, cause his teeth to chatter.

Meanwhile, Belinda dons a lovely pale blue dressing gown and wanders round the room looking for the nurse's address card, which she thrusts into his hand, and falls to hugging the bed-post and moaning softly.

"Do, do, hurry," she says plaintively. "Bike as far as the cabyard, and then go for nurse, and wake cook and tell her to come and light the fire."

He does so, but once on his machine scorches madly along until he reaches the nurse's abode, where he pulls the bell so vigorously it is a wonder it does not break. A window flies up above his head and he makes known his want.

Mrs. Gamp demands severely "whether a cab is coming for her?"

"Oh, by Jove!" he ejaculates, "I forgot all about it, what shall I do?"

"Why, go back at once, and send one as soon as you can, and see it start out of the yard," she adds significantly.

This he does and then routs out the doctor, who inquires down the speaking tube "Whether the nurse is there?" and on hearing she is not mumbles something uncomplimentary for he has only just been back in bed ten minutes from a similar business, and feels it rather hard lines to be called up again so soon. Mr. Younghusband speeds home, and he and Belinda sit hand in hand before the now cheery fire until the nurse arrives, who soon divests herself of bonnet and cloak, and appears dark-eyed, slim, and dainty in her lilac gown and snowy linen, much to the approval of the young man who had looked forward to the typical Gamp. But he rather shrinks under her severe look when he announces he has already been for the doctor.

"Oh, poor doctor," she says regretfully, "I don't think we shall want him just yet. What a pity!"

However, he presently arrives, and kindly reassures Belinda that all is going very well, and departs until he is really needed. And now nurse tries to send Mr. Younghusband off to bed, elsewhere, with the assurance she'll be only too glad to call him when needed. But not a bit of it he declares he couldn't possibly sleep a wink, and insists on installing himself at the far end of the landing, in a huge, padded, creaky wicker chair, which, with some cushions and a big rug, he hauls upstairs for the purpose, and where he squeaks and wriggles about in it, until at last he falls fast asleep until 7 A.M., when he is sent off for the doctor; and then after this he has a very tiresome wait, for they shut themselves in, and he anxiously speculates as to how his beloved Belinda is faring, and once or twice when the nurse passes him catches hold of her apron and detains her asking insane questions, and once he rushes downstairs to tell the cook to order lots of extra milk, "for the little beggar will be so hungry when it comes."

Presently he is prevailed upon to descend to breakfast, and is in the middle of that meal when he hears a curious small wailing in the room above him. Upstairs he bounds, three steps at a time, and raps briskly at the door.

Nurse opens it an inch. "Fine boy," she says, and shuts the door immediately. In less than five minutes he is back again handing in a dainty silver tray with three glasses and an opened champagne bottle on it.

"I'm sure you all want it" he begs persuasively, and so she takes it in to keep him quiet, and he fondly imagines his Belinda is having a glass to "keep her up."

After a while the doctor descends to a well-earned breakfast, and sends him upstairs just for two minutes, and when he enters he makes straight for the bed, falling on his knees beside it, and nurse strolls to the window and takes a long look down the street while they enjoy one of life's supremely happiest moments of joy over their first-born.

### To Nurses.

We invite contributions from any of our readers, and shall be glad to pay for "Notes on News from the Nursing World," or for articles describing nursing experiences at home or abroad dealing with any nursing question from an original point of view according to length. The minimum payment is 5s. Contributions on topical subjects are specially welcome. Notices of appointments, letters, entertainments, presentations, and deaths are not paid for, but we are always glad to receive them. All rejected manuscripts are returned in due course, and all payments for manuscripts used are made as early as possible after the beginning of each quarter.

### A Visit to the North Staffordshire Nurses' Home.

BY OUR COMMISSIONER.

The progress of the nursing movement is attested in many ways, and perhaps in none more conclusively than in the improved provision which is made for the accommodation of nurses. The provinces are successfully vieing with the metropolis in this respect, and if there are nurses in London who suppose that they have a monopoly of comforts, they would be speedily convinced to the contrary if they visited such a Home as that which has lately been opened under the auspices of the governors of the North Staffordshire Infirmary and Eye Hospital.

### The Site of the Home.

The Potteries themselves are not beautiful, but even the smoke of Stoke-on-Trent does not spoil some of its delightful environs, and Hartshill, on the crest of which the North Staffordshire Infirmary stands, is high above the town. The new Nurses' Home is even higher than the Infirmary, and commands from its windows and grounds a lovely view of well-wooded undulating country; while on a clear day the hills of North Wales are clearly visible in the far distance. It may be remembered that the Home was built as a Coronation Memorial, and it is, in fact, known locally as King Edward VII. Home for Nurses. The account which I am able to give will show that the promoters planned and have erected an institution worthy of the name, and that very good value has been obtained for the substantial sum of £10,000 which has been expended upon it. In the absence from Hartshill of the Superintendent of Nurses, Miss H. L. Pearse, on the occasion of my visit, I was received by the Home Sister, who conducted me over the building, and also through several wards of the infirmary, with which it is connected by a covered way of considerable length.

### The Sisters' Sitting-room.

Having expressed my admiration for the tennis-lawn and grounds, I asked the Home Sister as we went round to point out the features of the various rooms. We started with the sisters' sitting-room. The floor is covered by Persian carpet, and the furniture is upholstered in green leather. There are occasional easy chairs, an extra couch, and three small tables. Quite a feature are the upholstered covered ottomans round the bay of the window, underneath which there is space for needle-work and books. This room contains a bookcase with the library books, in reference to which the Home Sister mentioned that a small subscription is paid. The over-mantel, bright with flowers, is in white wood, the fireplace is tiled, and the fire-irons are copper. There are curtains round the double bay, and there is also a side window. A slow-combustion stove is used, and a serving hatch, with double doors to put the tea-trays in the kitchen, completes the list of conveniences.

### The Box-room and Visitors' Room.

On the ground-floor is the box-room, with a cemented floor for heavy boxes, and double shelves for light luggage. No boxes are therefore allowed in the bedrooms to disfigure the tops of the wardrobes. The pantry is close to, with tiled floor, shelves, and cupboards, while next to this is the large kitchen, with a special range that requires only the smallest quantity of fire. Next we went into the visitors' room, carpeted with Brussels, and containing easy and other chairs, polished table and tapestry cloth. A door in this room leads to a verandah which overlooks the garden. The library, with similar furniture, is adjoining, but here there is a book-case filled with medical books, the gift of the late house surgeon. These are lent to the nurses.

### The Probationers' Sitting-room.

A special feature of all the rooms is the ventilated boards which I noticed in the probationers' commodious sitting-room. The furniture includes an upright piano, a Chesterfield couch behind the door, easy leather and wicker chairs, rocking chairs, a tapestry upholstered chair, a number of small tables, and ottoman boxes round the double-bay windows. The floor is covered with green Brussels carpet.

"The sisters, when off duty, are supplied with tea, on giving due notice; and the probationers," said the Home Sister, "are allowed to make tea in their sitting-room when they are off duty, providing that they supply their own teacups, which are kept in the ottomans. Hot water and milk can be obtained from the kitchen.

"Are they allowed to have any other meal here," I inquired.

"They may have supper brought up by their fellow-nurses when they are off duty. They may also have breakfast in bed when they have their day off. In that case they are waited upon by the servant in the Home, and special crockery is provided by the infirmary. No food, except breakfast, is permitted in the bedroom, and no spirit stove is allowed for any purpose. The probationers can remain in the sitting-room until 10 A.M , and in one another's bedrooms until 9.30, and lights are out at 10.30."

### Regulations and Training.

This seemed an opportune moment to ask a few questions about the regulations and training. As to the hours on duty, the Home Sister informed me that the probationers are called at 6, breakfast at 6.30, enter the ward at 7, have dinner at 1.30 in the infirmary, and leave the wards at 8. They have 12 hours off duty during the week, one long day during the month, half a day each fortnight, and three weeks' holiday during the year.

"The period of training is three years?" I asked.

"Yes, and two examinations have been made compulsory by the present Superintendent of Nurses. We have two certificates, one of the ordinary character which is given at the end of the three years. If a nurse does not pass the final examination she has a certificate saying that she has been in residence and has been a good nurse. But very few of the latter certificates are issued."

"I suppose you have the usual lectures?"

"The Superintendent of Nurses gives a course for the first year, and the resident physician and surgeon the third year. Attendance by the probationers at these courses is compulsory. Miss Pearse hopes soon to have a preliminary examination before admission."

"What about salaries?"

"No salary is paid the first year; £10 the second year; £18 the third, and if a probationer stays on as staff nurse, she is paid at the rate of £25 a year. The sisters are almost invariably promoted from the staff."

### The Sisters.

"How many sisters are there?"

"Twelve. Every sister has to work for six months on trial as a staff nurse before being promoted. The sisters are called at 7, breakfast at 7.30, enter the wards at 8, and are in charge until 9. They have an entire day off every month, half a day every week, and are off duty for some time every other day. As to holidays, they have three weeks in summer and one in winter. The salaries are from £30 to £35, and uniform is provided. There is a night superintendent, a theatre sister, and home sister."

### The Bedrooms.

The Home Sister, who was herself trained at Leicester Infirmary, and has had 12 years' experience, including a year at one of the fever hospitals under the Metropolitan Asylums Board, then proceeded to show me some of the bedrooms. Those of three ward sisters, as well as two sick-rooms, are on the ground-flour. The furniture is similar:—Blue carpet on the centre of the floor, which is polished all round, a mat in front of the dressing-table, a black spring-bedstead, a corner washstand with marble top, tiled back, and splash curtain; dressing-table and chest of drawers combined; small shelves for trinkets; special long toilet-mats made to fit; corner wardrobe with long mirror, and box inside for hats; towel-rail, Japan coal-scuttle, and corner linen-basket. The whole of the linen-baskets were made by the children of the Cripples' Guild, through the good services of Mrs. Coghill, the ex-President's wife.

Even now, however, we had not finished our inspection of the ground floor, which also includes an excellent store closet, a lavatory and bath-room, large linen-room, an admirably-appointed cloak-room with a small box for shoes and a peg for clothes for each nurse; a cycle room which holds a number of vehicles and opens into the grounds; a lobby with housemaid's pantry and bath-room; a pleasant hall with fireplace, the night superintendent's sitting-room, and the home sister's sitting and bedroom.

### The First and Second Floors.

Ascending the staircase, we looked into one of the six bedrooms belonging to the sisters. They are equipped like the rooms below. Quite a feature is the long wide corridor, coloured green, heated with radiators, lighted by electric light—like the whole of the home—at each end being a white enamelled orderly-box for rubbish. On this floor there are 15 probationer's bedrooms, the furniture being, to a large extent, the same as that in the sisters' rooms, except that it was not made to fit into the corners and was not so costly. A noticeable small article is a brass guard for the fireplace, which is in every room, though a fire can only be lighted by special permission. Every room has been so constructed and so furnished that there is not a corner unoccupied; and thus, while every inch of space is turned to good account, there is the maximum of air. Altogether, the Home Sister stated, there are 57 bedrooms in the home. These include three for the maids who sleep on the second floor. Here, too, divided by double baize doors, are 14 bedrooms for the night superintendent and nurses, two bath-rooms, and lavatory. The arrangements for ensuring quiet for the night nurses who sleep during the day are all that could be desired.

### The Infirmary.

As we made our way into the infirmary, the Home Sister mentioned that the Superintendent of Nurses, whose apartments are, of course, in the main building, presides at the sisters' dinner, while she presides at that of the probationers. The night superintendent presides at the probationers' breakfast, and she and the Home Sister alternately at the breakfast of the night nurses and the probationers' supper. In order to give an idea of the work of the nurses in the infirmary I noted a few particulars. There are 220 beds, of which 180 are generally full. Four large wards for women—gynæcological, ophthalmic, medical, and aseptic—form one block; and there are four in another block for males, one being an accident ward, with a receiving-room attached, and another for cases of stricture. Adjoining the accident ward is a balcony for open-air treatment for cases of tuberculosis, and here the patients sleep in all weathers. The medical wards are on the first floor—two for men, one large and one small for women. At the entrance, with a sister in charge, is a children's ward with 16 beds. There is a small block for special cases, and an isolation block with four beds. The operating-room is large, with tiled walls, and furnished throughout on up-to-date lines; and, finally, there is a nicely appointed chapel which is used for Sunday and daily service. The varied nature of the cases, many of them very serious, and almost all acute, must enable the nurses of the North Staffordshire Infirmary to gain extremely valuable experience; and their home, whose exterior is as unpretending as the interior is perfect, must be a constant source of gratification to the entire staff.

### A Question of Colour.

BY A BRITISH NURSE.

Not long ago I was consulted by a friend as to the wisdom of her becoming a candidate for a recently advertised vacancy at a newly-founded "home" under the direct control of an important public body; the work of this home, I may add, is in the main of a reformative character, but training in nursing is an essential qualification. I advised my friend to apply for the vacant situation, and we awaited with interest the arrival of the official reply. This reply of course took the shape of the inevitable "form" to be filled up; the questions asked were perhaps somewhat more searching than is generally the case, but not sufficiently so to call for remark excepting those which required an answer as to the colouring of hair, eyes, and complexion of the applicant. As the form bore at the top the awe-inspiring information that all appointments were subject to the approval of a Cabinet Minister I could not pursue with any real conviction my initial line of reasoning and conclude, as I was at first prepared to do, that the officials had more available space at command than queries with which to fill that space. I therefore made an effort to discover the true inwardness of these quite exceptional questions. I have had a rather large experience of being consulted over "forms" of various kinds, and never before had I seen a description demanded for individual "colouring"; it struck me as odd, and I determined to consult a friendly doctor who was so fresh from a big hospital that I knew his theories had not become obsolete. I laid both the facts and the "form" before him, and explained that I found myself labouring under two distinct difficulties. In the first place my friend had asked me to be quite frank and to tell her how she could accurately describe her colouring. I could only say, "you are simply mouse-like: hair, eyes, everything, and you can't put *that* on a form; true it certainly is, but it might, put in that way, cause you to lose an appointment." My doctor friend was not helpful on this point and advised a reply of the safe kind gipsy fortune-tellers are fond of giving when they are asked specific questions as to the colouring of unknown rivals; they vaguely answer "he or she is *between-coloured*."

On the next point of difficulty I had a more direct opinion. "Oh! dear yes," the young doctor said, "I thought everybody knew that in positions in which the possession of moral qualities are of first importance an immense amount of consideration is paid to this question." "Surely that is very empirical," I rejoined with some heat, "and quite as dangerous as the old idea that phrenology is of any real value in deciding on character?"

But the doctor was firm and told me that I ought not to live entirely out of touch with modern views; and added that a great deal of attention and study showed that complexions, etc., were facts of moment. I desired above all things to be practical, and urged upon him the need to be more explicit. "You mean to imply, do you not, for instance, that the saying 'fair people are treacherous' is correct?" I asked. "More or less," he answered, "there can be no question in the mind of anyone who has studied the subject that people with dark colouring and dark eyes are likely to be more trustworthy than fair people. The post which your friend wishes to fill is one that needs the very highest moral qualities and as questions are asked it is clear the replies will form a factor in considering her application." I fear I grew hot at this stage, and said various things about modern theories which were not quite respectful to their makers, and thereafter the discussion lapsed into frivolity. I walked home thoughtfully, and to bring to a close the personal part of the episode, I may say here, that though the form was carefully and respectfully filled up with a view to the important personage's approval, and although the "mousiness" of my friend's colouring was indicated with a careful choice of words, nothing more, alas, was heard of the application! From this fact I wonder if it is safe to deduce a generalisation, and to say that "between-coloured" people are not eligible for these particular appointments? I can imagine no other reason, as my friend's qualifications in every other direction [are above reproach—as was duly proved by recent and excellent testimonials. I was very sorry for the sake of the institution and proceeded to make further deductions with a fervour born of resentment.

If this idea were to spread what hope is there for some of us? Are we to have a return of the easy classification of the old melodrama and know what amount of innocence or guilt is intended to be conveyed when the dark or fair heroine appears? Perish such a prospect! I called up in the dark watches all my best tried fellow-workers, and decided that a more unfair rendering of character could not be devised than a tendency to differentiate in respect of colouring. One of the finest and truest workers I recalled had in the question of eyes always puzzled me sorely, for I was aware they were different in varying lights, and I was not sure that they quite matched! And yet with "a form" before one (and a looking-glass) what would truth not demand? Question: "State colour of eyes." Answer: as one poet says of the sea—"The greyest of all things green, the greenest of all things grey"; or "one light grey, the other pale green." In all seriousness will not there be a tendency in future if this plan is pursued at all widely, that committees may soon lay too much stress on what is still in many minds a matter of accident, and thus prejudice unfavourably the chances of many candidates? If there is any solid foundation in my medical friend's views, the question has, however, a wider basis] in fact than I at least was prepared to admit without more knowledge. If there is truth in the view that dark people are as a rule more truthful, loyal, and sober, it is surely very desirable that all of us who have the duty of choosing workers should have access to the facts on which such an assumption is based. If, however, the question is still only under discussion I think the initiative in asking such questions should not be taken by a great public body employing as it does hundreds of workers. The matter should be considered scientifically, and a decision arrived at only on clear and tangible grounds.

### The Central Midwives Board.

The following is the draft "Scheme of Examinations," which will come up for final revision by the Central Midwives Board in the autumn.

### 1. Examiners.

(1) A list of examiners, both for London and the Provinces, shall be prepared by the Central Midwives Board from those who are willing to serve and act, when required, by the Board. This list shall be subject to annual revision by the Board. Before such a list is formed, applications for the post of examiner shall be invited by advertisement. (2) Every examination shall be partly oral and practical, and partly written, and shall be conducted by not fewer than two examiners. (3) No examiner shall examine his own pupils. (4) The first examination shall be held in May 1905. (5) Thereafter the examination shall be held four times a year, or oftener if necessary, in London and the Provinces, simultaneously, on the same papers. The Board reserves to itself the right to appoint one or more qualified medical practitioners as visitors of the examinations, both London and Provincial, when it appears desirable. (6) The first Provincial Centres shall be:—*a.* Bristol; *b.* Manchester; *c.* Newcastle-on-Tyne. (7) A paper of directions to examiners shall be drawn up and issued to each examiner on his appointment. (8) The remuneration of an examiner shall be at the rate of 7s. for each candidate examined.

### 2. London Examiners.

(1) The examiners, both London and Provincial, shall be invited to meet at the offices of the Central Midwives Board as often as may be necessary. (2) The arrangements for the examinations shall be made by the secretary of the Central Midwives Board, with such extra assistance as may be necessary. (3) The duties of the London examiners shall be:—(*a*) To consider examination questions suggested by provincial examiners. (*b*) To set all the papers of examination questions both for London and the Provinces. Two of the examiners, with the assistance of one of the medical members of the Board to be appointed for the purpose, shall undertake this duty in rotation. The remuneration of the examiners shall be two guineas each. (*c*) To conduct the examination, written and oral, of all candidates presenting themselves for examination in London. (*d*) To report to the Central Midwives Board the result of each examination held in London.

### 3. Provincial Examiners.

(1) The examiners appointed by the Central Midwives Board to conduct the Board's examinations in a provincial centre shall assist the Secretary of the Board in making the necessary arrangements for the examinations. (2) One of the Examiners shall be responsible for reporting the result of each examination to the Central Midwives Board.

### 4. Directions to Examiners.

(1) The paper shall consist of not fewer than six questions, and the time allowed for answering shall be three hours. (2) Notes should be taken, either by the examiner himself or by a colleague, of the subjects upon which the candidate is questioned in the oral examination. (3) Fifteen minutes should be considered an average period for the oral examination of each candidate. (4) Examiners should avoid questioning candidates as to where and by whom they were trained, so as to avoid any suspicion of bias.

### To One of Many.

You asked me once to write some rhymes for you,
Whose mission is beside the beds of pain;
Carry that gentle look, sweet eyes of blue,
From this your youth, till youth and life shall wane.
The kindly touch, the sharp word left unsaid,
The doing deeds that others might despise—
So shall I think of you when years have fled,
O sweet blue eyes!

You never were so tired but that you
Had gracious words and thought for each one's care,
You had a gentle look, O eyes of blue,
You often bore what others would not bear,
Th' impatience of the over-burdened brain,
The fretful wants expressed in fretful guise,
It was from YOU, faint hearts drew hope again
O brave, blue eyes!

'Tis not the heroic acts that people do
(These stir the blood but scarcely touch the heart),
It is the little tender deeds and true,
That from the memory never can depart.
Identified Himself with each sad bed,
"Ye did it unto Me," the Master cries,
Be ever patient then, wise heart and head,
And dear blue eyes!

LILIAN CLAXTON.

### Everybody's Opinion.

### THE GUILD OF ST. BARNABAS.

MISS C. J. WOOD, secretary of the Guild of St. Barnabas, writes: With reference to your article on the Guild of St. Barnabas, in which you say "its offices are in Brooke Street, Holborn," please allow me to state that we have no offices, and that all business is transacted at my address, The Nurses' Hostel, Francis Street, London, W.C.

### THE HOSPITAL DUNCE AND THE INSTITUTION FRAUD.

BESSIE CAWOOD writes: Those who object to State registration with a qualifying examination, on the ground that a nurse's character is of more importance than her technical knowledge, seem to forget that the public can judge of the one but not of the other. People can watch a nurse as they would a governess or servant, and find out for themselves if she is "good-tempered, sympathetic, and patient." They cannot tell if she knows her work. That "the complaints one hears of nurses . . . . are more often due to her conduct than to lack of skill" only shows the truth of this. The whole line of argument is like that of an old-fashioned schoolmistress, who veils her dislike at having to conform to a regulated standard of teaching, by assuming fears for her pupils' moral qualities. A woman may not act as a midwife until she has been tested by a disinterested authority. It seems to be generally admitted that it is possible to make sure that she at least possesses the minimum of knowledge which makes it safe for her to practise. It is hard to see why a like test should not be applied to professional nurses. It is true, as one objector urged, that the technical knowledge necessary for a nurse in order to safeguard the public is so small that the examination would be childishly simple. Nevertheless many nurses leave hospital after a three years' training without possessing it in a workable form. The value of the examination would not only be in the test, but in the preparation for it. Governing bodies would have to make sure that the matrons they appointed were really capable of giving systematic teaching. The three years' certificate from a recognised training school would have its present value, but the public would be protected from the hospital dunce as well as from the institution fraud.

### GAMP MIDWIVES.

"DISTRICT NURSE" writes: Having been a Queen's nurse for several years, and holding the L.O.S. Certificate, I have been greatly shocked by the terrible methods of the Gamp widwives in this town, in which I am now working as a district nurse, though not as a midwife. I am not surprised to hear so many poor women trace a life-time of suffering and ill-health to their confinements, when the usual methods of these women is to have the baby born on the floor, and afterwards drag the poor mother into bed! Again on the third day after confinement, it is the custom to take the mother out of bed to have the bed made! As to the dirt, it is simply indescribable. I have known several cases in which the woman has had unchanged for days the clothes in which she was confined, which have hung round the poor woman as hard as a board. More details are not necessary, I think. In 1910, we hope, happier days are to dawn for the unfortunate mothers, but meanwhile may I offer a suggestion for making the best of a bad bargain? Would it not be possible to employ a qualified midwife in every town to give practical instruction, in at least the monthly nursing part of a midwife's duties, to those women who are provisionally certificated on the ground of having practised midwifery for one year and being women of good character? I do not think that lectures would be much good, but I do think that something might be done by a good and properly qualified midwife going round to, say, two confinement cases with each of those women, and showing them how to manage at that time and also in the after-nursing of the patient. I am quite aware that there might be many who would resent the idea of being taught, and the great difficulty in the way would be the conceit which usually accompanies such ignorance. To meet this, the reception of such instruction would have to be made compulsory by those in authority, who would require to issue a command to each of these women to avail themselves of the instruction offered.

### RED TAPE IN THE WARDS.

"LOOKER-ON" writes: Although I fully sympathise with the tone of "Bet's" letter, it is only fair, as the writer of "Red Tape in the Wards," to say that the habits of which she complains do (or a short time ago did) exist. I have seen patients' milk habitually skimmed for "sisters' tea"; the patients were not allowed to toss and turn so as to disturb the immaculate neatness of their bed; the nurses were not allowed to dry the babies' "doubles" by the fire for fear of "spoiling the look of the ward"; patients were rebuked for requiring necessary attentions when the doctor was expected; a demand for a toothbrush was met with surprise. The hospitals to which I refer are excellent institutions, and no doubt many of the customs I have mentioned have disappeared lately in the wake of modern improvements. But the important question is: Does the routine spirit, unredeemed by sympathy, remain in any hospital, large or small? I went for my training when I was no longer young; my sympathetic and critical faculties were, consequently, fully awake. Nevertheless, I had sometimes to pull myself up in the midst of the exhilarating swing of smart ward work, and say, also to myself: "Look here; it really is better to be dropped on for not dusting properly than to refuse to get that poor chap some 'lemon drink!'" And I often wondered what the result would be on young girls, who could not be expected to understand the sick person's point of view. "Tout comprendre c'est tout pardonner." But until time and suffering have revealed to us the truth of this axiom, many of us to whom tenderness does not come intuitively, must be taught sympathy. Wards must be spotless; nurses must be quick, and the ultimate object in being so is the welfare of the patient. I really believe it to be necessary to keep this fact before the minds of young probationers. If sisters and older nurses would only explain why beds are made in a way that appears only to inconvenience the patient; if they would only squash any tendency to complain angrily of that "awful No. III., who is always being sick," the hospital would be a better school for sympathy than it at present is.

### Appointments.

[No charge is made for announcements under this head, and we are always glad to receive, and publish, appointments. The information, to insure accuracy, should be sent from the nurses themselves, and we cannot undertake to correct official announcements which may happen to be inaccurate. It is essential that in all cases the school of training should be given.]

Braintree Union Infirmary.—Miss Grace M. Lloyd has been appointed superintendent nurse. She was trained at Sunderland Infirmary, and has since been sister at Shoreditch Infirmary, superintendent nurse at Hitchin Infirmary, head nurse at Lewes Infirmary, and theatre sister at Tottenham Accident Hospital. She holds the London Obstetrical Society's certificate, and that of Apothecaries' Hall for dispensing, and in January 1904 she won a silver cup for "Hygiene" at École de Science, Paris.

Children's Hospital, Birkenhead.—Miss Annie Lennox has been appointed sister. She was trained at Northampton General Hospital, where she also held the post of sister.

Hendon Sick Asylum.—Miss Alice Wilson has been appointed charge nurse. She was trained at Scarborough, and has since been charge nurse at the Victoria Park Hospital for Diseases of the Chest, London.

Mansfield Woodhouse Medical Hospital and Convalescent Home, Mansfield, Notts.—Miss H. E. Yardley has been appointed matron. She was trained at the General Hospital, Rotherham, where she was afterwards night superintendent. She has since been sister of the surgical wards and casualty department at the General Infirmary, Burton-on-Trent; sister of the surgical, ophthalmic, and special wards, and matron's assistant at the North Riding Infirmary, Middlesbrough; and sister of the children's department at the Southern Hospital, Manchester, where she has also taken matron's holiday duties.

The David Lewis Manchester Epileptic Colony, Warford.—Miss Alice Stapeley has been appointed matron. She was trained at the North-West London Hospital and Lambeth Infirmary, and has since been charge nurse at the Western Hospital, Fulham.

### TRAVEL NOTES AND QUERIES.

By our Travel Correspondent.

A Holiday in Belgium for Five Pounds (Jean).—You would, I think, like Bruges, for it is full of varied interest. At the Hôtel Panier d'Or, in the market place, you would not pay more than 7 francs per day if you had a small room high up. For a week the cost would be just under £2. The journey from London to Ostend, second-class return, is 9s. 6d., *via* Tower Bridge. Write for dates of sailing to the General Steam Navigation Company, 33 Great Tower Street. Boats go three times a week. The rail from Ostend to Bruges is short and inexpensive, perhaps 1s. or 1s. 6d. The excursions round Bruges are easy and cheap. Hotel tips for a week will come to about 8 francs.

Rules in Regard to Correspondence for this Section.—All questioners must use a pseudonym for publication, but the communication must also bear the writer's own name and address as well, which will be regarded as confidential. All such communications to be addressed "Travel Editor, 'Nursing Section of *The Hospital*,' 28 Southampton Street, Strand." No charge will be made for inserting and answering questions in the inquiry column, and all will be answered in rotation as space permits. If an answer by letter is required, a stamped and addressed envelope must be enclosed, together with 2s. 6d., which fee will be devoted to the objects of "The Hospital" Convalescent Fund. Any inquiries reaching the office after Monday cannot be answered in "The Hospital" of the current week.

### The Nurses' Bookshelf.

A Nursing Guide. (Second series.) (London: Ash and Co. Ltd., 42 Southwark Street, S.E. 1904. Octavo, p. 141, advt. p. xx., paper boards 1s. 6d.)

This publication, issued under the auspices of Guy's Hospital, is described on its title-page as a "Nursing Guide, Handbook of Nurses' League, and Register of Nurses Trained at Guy's Hospital." The compilers have endeavoured to produce a book which shall be useful to both trained nurses and intending probationers. The resulting pot pourri is not altogether happy, and we would suggest that a future edition should either ignore the would-be nurse, giving additional space to the cooking receipts and other valuable nursing memoranda, or else be amplified into two volumes, each addressed to its own class of readers. The book would also be much improved by the use of better printing and paper and the addition of an index. The articles on private and district nursing are instructive, and many nurses will read with interest the new regulations of the Nursing Services and Midwives Act. In "Useful Nursing Information" the private nurse will find much to refresh her memory; the sections "Urine Testing" and "Poisons and Antidotes" being particularly clear and good. The perusal of "A Nursing Guide" shows clearly how fully the authorities of Guy's Hospital recognise the advisability of varied and healthy recreation for their nurses. Every accepted probationer must become a member of the Nurses' League, the rules of which include regulations for a tennis club, a lending library, a choral society, a debating society, a swimming bath where certificates for proficiency may be earned, and a cycling club, the machines of which can be hired at a penny for three hours! There is also a cottage where members may store their own cycles and spend their off-duty hours, and where those for whom the hospital provides board may obtain any meal for the modest charge of 1d. for service. The Register of Nurses, Promotions, and Obituary Notices, together with the "Nursing News" at the end of the volume, will be of great interest to all old Guy's nurses, and we should think that this little annual will do much towards helping them to keep in touch with the old hospital of which they are so justly proud.

The Englishwoman's Year Book and Directory, 1904. (Adam and Charles Black. 2s. 6d.)

This admirable guide to women's work and interests should be in the hands of all working women and of all who desire to understand what they are doing in the world. The volume, which is appropriately edited by Miss Emily James, organising secretary to the National Union of Women Workers of Great Britain and Ireland, contains in a convenient compass a vast amount of useful information respecting the professions in which women are engaged, the charitable institutions which exist for their benefit, the religious organisations designed to promote their welfare and gain their support. Not the least valuable feature is a directory of prominent "working women," which is handy for reference to all sorts of people.

Rudderless Ships. By Airam. (Henry Drane. 6s.)

In this story the author attempts to make the marriage of two persons in whose family, on the wife's side, there was an hereditary taint of insanity, an object-lesson on the imprudence of such a union. Every known calamity follows the marriage of Herbert Mortimer and Edith Weston. In the epilogue "Airam" sums up her views on the question. The following is an extract:—"If an hereditary mental instability is known to exist in a family, then wise is the man or the woman who decides to let that particular line die out. Let them cultivate assiduously any special talent they may have, and thus aim at making for themselves a name. With the high mental capacity many of them possess this might be well achieved."

### Echoes from the Outside World.

### The King at Marienbad.

The King reached Marienbad at five o'clock last Friday afternoon, and was received at the railway station by the British Ambassador, with whom he drove in a motor-car to the Hôtel Weimar, where he has a suite of apartments on the first floor. The Burgomaster of Marienbad has posted notices throughout the town requesting the public during King Edward's visit to abstain from doing anything which might cause discomfort or embarrassment to their distinguished guest, such as unduly crowding around him. As a result, on Saturday his Majesty was able to walk about and sit down comparatively undisturbed by curious spectators. After breakfast on Saturday the King took a bath in the chalybeate and saline waters of the Ferdinands-brunnen, in the New Badhaus; lunched with Prince Ferdinand of Bulgaria at the Waldmühle Restaurant; and dined with Count Mensdorff. On Sunday morning he attended the service in the English church, the small congregation present consisting almost entirely of English visitors. On Monday the King gave a dinner party, at which the Duke of Teck was present, and on Tuesday the Emperor Francis Joseph visited Marienbad. The Emperor was met at the station by King Edward, who wore his Austro-Hungarian field-marshal's uniform, with the ribbon of the Order of St. Stephen, while the Emperor returned the compliment by wearing his British field-marshal's uniform, with the ribbon of the Garter. The Emperor on alighting not only shook the King by the hand, but kissed him on both cheeks. Their Majesties lunched and dined together, King Edward being the host, and at dinner they toasted each other amid the enthusiastic applause of the invited guests. At night the streets of the town were brilliantly illuminated. The Emperor left Marienbad on Wednesday morning for Karlsbad.

### Birth of an Heir to the Russian Throne.

On Friday last week the birth of a son and heir to the Tsar Nicholas II. took place. The joyful event was announced to the inhabitants of St. Petersburg by a Royal salute, which many at first interpreted as a sign that the Russian squadron had defeated the Japanese at sea. But a little later policemen visited the watchmen of the houses and instructed them to hoist flags and prepare illuminations to celebrate the advent of a Tsarevitch. At the same time tradesmen were exhorted to give their clerks and salespeople a few extra free hours in the evening. The foreign ambassadors and ministers called at the Foreign Office to offer their congratulations to the Tsar and the Tsaritsa. The birth of the little prince, who has already been given the name of Alexis, increases the Imperial family to five. The other four children are daughters, and were born respectively in 1895, 1897, 1899, and 1901. The Grand Duke Michael, hitherto the heir presumptive, now descends to the rank of an ordinary Grand Duke, and the infant just born receives the full titles and money allowances of which the Grand Duke Michael possessed only a part.

### Great Naval Battles in the Far East.

It was officially announced on Monday that a great naval engagement, lasting five hours, took place on Sunday morning in Korea Strait, between three cruisers of the Vladivostock Squadron and some Japanese warships. One Russian cruiser was sunk and two others returned northward badly damaged. It is also officially reported that the Russian battleship *Czarevitch*, from Port Arthur, arrived at Tsing-tau almost a wreck, having had a running fight with the Japanese Fleet, during which one Russian admiral was killed. The Mikado has signified his wish that all non-combatants shall be allowed to leave Port Arthur by way of Dalny before the general assault commences. A further report from Admiral Kamimura respecting his action with the Vladivostok cruisers states that all three of the Russian cruisers caught fire during the action, and appeared to suffer heavily. Eventually, two of the Russian cruisers fled northward, leaving behind the *Rurik*, which shortly afterwards sank. The whole Japanese squadron hastened to rescue the drowning Russians, and picked up about 600 of them.

### The Late M. Waldeck-Rousseau.

A telegram was sent on Friday last week by King Edward from Marienbad to the British Embassy at Paris, requesting that the sincere condolences of His Majesty might be conveyed to the family of M. Waldeck-Rousseau, with assurances of the grief which the loss of the eminent statesman had caused him. The King was represented at the funeral ceremony on Saturday by M. de Bunsen, British Chargé d'Affaires. A State funeral was offered to the widow of M. Waldeck-Rousseau and refused, partly because she desired a simple ceremony, and partly because M. Combes had notified to her that such obsequies would be of a strictly civil character, without any religious service. More than 1,500 persons defiled before the grave, and sprinkled the coffin with holy water. M. Waldeck-Rousseau, who died at his country house at Corbeil last week, after a second operation, at the age of 58, was ex-Premier of France, and her most illustrious statesman. He began life as a lawyer, and entered the Cabinet of M. Gambetta in 1881, as Minister of the Interior. In 1893 he defended M. de Lesseps in the action arising out of the Panama scandal. In June 1899 he became Premier, relinquishing an income of £20,000 a year in order to undertake the office.

### Births, Marriages, and Deaths.

The 65th annual report of the Registrar-General dealing with the births, marriages, and deaths in England and Wales during the year 1902 has just been issued. The births registered were 940,509, the rate per 1,000 of the population being the same as in 1901. The male births were to the female in the proportion of 1,039 to 1,000, the average proportion in the preceding decennium having been 1,036 to 1,000. The deaths registered during the year numbered 538,558, and were in the proportion of 16·2 per 1,000 to the population of all ages. Never since 1837, when civil registration was established, has the English death-rate been so low as this. The number of marriages was 261,750, of which 173,011 were solemnised according to the rites of the Church of England, the proportion, however, being the lowest on record. Of the men who married, 909 per 1,000 were bachelors, and 91 per 1,000 were widowers. Of the women 932 per 1,000 were spinsters, and 68 per 1,000 were widows. Of the persons who married in the course of the year, 479 are described as having been previously divorced, and in 24 cases divorced men married divorced women. Among adults 1·15 per cent. of the husbands and 1·40 per cent. of the wives were indefinitely described as "of full age."

### Heroism and Bravery.

On Tuesday an inquest was held on the bodies of Lady Hilda Maud McNeill and Master Glyn Pritchard, who were drowned on Monday in the river Taw, at Fremington, near Barnstaple. Lady Hilda, as the evidence showed, lost her life entirely in consequence of her effort to save young Pritchard, who had entered the water to bathe before her, but both were carried away by the strong ebb tide. She was a sister of the Earl of Stradbroke and was only 37. Miss May Pritchard, a girl of 13, was complimented by the jury on the gallant endeavour she made, by wading into the water, to rescue Lady Hilda and her brother.

### Notes and Queries.

**FOR REGULATIONS SEE PAGE 143.**

*Entering an English Hospital.*

(150) I am not a native of England, but should like to enter an English hospital. I have had nine years' experience in two hospitals abroad. Will you tell me how to proceed?—*M. S.* (*Hull*).

If you wish to go in for an English training, buy "The Nursing Profession: How and Where to Train," price 2s. You will find full details therein; or advertise.

*Pelvis. Skull.*

(151) Can you inform me if it is possible to hire a pelvis and fœtal head to use in coaching for the L.O.S.? What is the usual price to give when buying one?—*L. P.*

Write to the Secretary of the Association for Promoting the Training and Supply of Midwives, 12 Buckingham Street, Strand, W.C.

*Insane Patients.*

(152) Would a lady experienced in asylums have much difficulty in getting a license to receive insane lady patients into her own house? Is the license expensive?—*C. M. H.*

Write to the Secretary of the Lunacy Board for England and Wales, 66 Victoria Street, London, S.W., for full particulars.

*Lecturers.*

(153) I shall be grateful if you would kindly tell me the best way to qualify as lecturer in hygiene and the care of infants. I have been a district nurse for 11 years, and during the last eight and a half years I have attended over 300 cases of midwifery successfully. Would these qualifications suffice to secure me a post as inspector of midwives under a public body?—*H. I. J.*

Write respecting the qualifications of health lecturer to the Secretary, the Sanitary Institute, Margaret Street, W.; and for particulars of midwifery inspectorships to the Central Board of Midwives, 6 Suffolk Street, Pall Mall, W.C.

*Uniforms.*

(154) Are there any restrictions regarding the wearing of uniforms by private nurses, and can badges be worn without registration?—*L. B.*

Every nurse is free to wear any uniform or badge excepting, of course, those designed and registered to denote any special body with which she is not connected.

*Noises in the Head.*

(155) Will you kindly tell me if —— is a safe person to apply to for treatment of noises in the head, and if not, could you tell me where to apply for advice?—*F. L. L.*

Your doctor is the best person to advise you. If it is a question of aural disturbance, aurists are attached to all the large general hospitals, as well as to the special Throat and Ear Hospitals.

*Address.*

(156) Will you kindly give me the address of the Rural Midwives Association?—*M. S.*

47 Victoria Street, S.W.

*Registered.*

(157) I shall be glad if you will kindly tell me how to become registered according to the new Act?—*A. B. M.* and *Brighton Midwife.*

See "How to Become a Certified Midwife," by Louise Appell. 1s. 1d. post free, Scientific Press.

*Vienna.*

(158) Will you kindly let me know if there is an English nursing institute in Vienna?—*Nurse E.*

We know of none.

**Handbooks for Nurses.**

"How to Become a Nurse: The Nursing Profession." 2s. net; 2s. 4d. post free.

"The Nurses' Dictionary." By Honnor Morten. 2s. post free.

"A Handbook for Nurses." By J. K. Watson, M.D. 5s. net; 5s. 4d. post free.

"A Practical Guide to Surgical Bandaging and Dressings." By W. Johnson Smith, F.R.C.S. 2s. post free.

"The Art of Massage." By A. Creighton Hale. 6s. post free.

"Preparation for Operation in Private Houses." By Stanmore Bishop, F.R.C.S. 6d. post free.

The above works are obtainable of all booksellers, or direct from The Scientific Press, Limited, 28 & 29 Southampton Street, Strand, London, W.C.

### For Reading to the Sick.

HOME INFLUENCE.

THE many make the household
But only One the home.

*Lowell.*

Sweet is the smile of home; the mutual look
Where hearts are of each other sure;
Sweet all the joys that crowd the household nook,
The haunt of all affections pure.

*Keble.*

The thought of the holy home at Nazareth is full of consolation and instruction. Its peace, its simplicity and charity, its daily round of humble toil, its conversation as of angels, all give us the ideal of a Christian home. There is a magic beauty in a true home, where all breathes love and good-will, where there is sympathy and thought for others. God's blessing rests upon it.

Has God included in His gifts to you a happy home, with father and mother, brothers and sisters, all united in true love and zeal for one another's happiness? If so, your illness will but endear you the more to all at home, and bring out in strong relief the love and interest that are felt for you. Let the thought of the sweetness of home be a support to you, and often it will pierce a cloud and allow light to be seen. By your patience under suffering promote the brightness of home, and value the opportunities for victory which your life at home daily offers to you. Small acts of generosity, of giving up your own will, your own opinion; little acts of obedience, of patience, of restraint in speech—these will daily come to hand, and, if rightly treated, then between your home and Nazareth there will be a close and beautiful resemblance.

I can pray; I can suffer; yes, and all for Jesus. This is solid, daily work, with an abundance of fruit. Can I do anything else? Yes, if I am true to grace, my sick-room will be a centre whence I may promote gentleness and patience. My life, then, even when I am ill, is full of opportunity! What joy so to think and know! My prayers and pains are an apostolate with world-wide fruits, and they mould me in such a way that a mission is open to me in my home. Blessed be Jesus Christ, whose school is at Nazareth!—*Anon.*

O near ones, dear ones, you in whose right hands
Our own rests calm; whose faithful hearts all day
Wide open wait till back from distant lands
Thought, the tired traveller, wends his homeward way!
Helpmates and hearthmates, gladdeners of gone years,
Tender companions of our serious days,
Who colour with your kisses, smiles, and tears
Life's worn web woven over wonted ways,
Oh shut the world out from the heart you cheer!
Tho' small the circle of your smiles may be,
The world is distant, and your smiles are near,
This makes you more than all the world to me!

*Lytton.*

